# Gastric Carcinomas in Young (Younger than 40 Years) Chinese Patients: Clinicopathology, Family History, and Postresection Survival

**DOI:** 10.1097/MD.0000000000002873

**Published:** 2016-03-07

**Authors:** Fan Zhou, Jiong Shi, Cheng Fang, Xiaoping Zou, Qin Huang

**Affiliations:** From the Gastroenterology Department (FZ, CF, XZ), Drum Tower Hospital Affiliated to Nanjing University Medical School; Pathology Department (JS, QH), Drum Tower Hospital Affiliated to Nanjing University Medical School, Nanjing, Jiangsu, China; and Pathology Department (QH), VA Boston Healthcare System and Harvard Medical School, Boston, MA.

## Abstract

Little is known about clinicopathological characteristics of gastric carcinoma (GC) in young (≤40 years) Chinese patients. We aimed in this study to analyze those features along with family history and prognostic factors after resection.

We retrospectively reviewed all 4671 GC resections (surgical and endoscopic) performed at our center from 2004 to 2014 and identified 152 (3.2%) consecutive young patients. Patient demographics, clinical results, family history, and endoscopic-pathological findings were analyzed along with the older (>41 years) GC controls recruited in the same study period. Clinicopathological factors related to postresection outcomes were assessed statistically.

The trend of GC resections in young patients was not changed over the study period. Compared to old GCs, the young GC cohort was predominant in women, positive family history, middle gastric location, the diffuse histology type, shorter duration of symptoms, and advanced stage (pIII+pIV, 53.3%). Radical resection was carried out in 90.1% (n = 137) with a better 5-year survival rate (70.3%) than palliative surgery (0%, n = 15). There was no significant difference in clinicopathological characteristics between familial GC (FGC, n = 38) and sporadic GC (SGC, n = 114) groups. Very young patients (≤ 30 years, n = 38) showed lower *Helicobacter pylori (Hp)* infection and significantly higher perineural invasion rates, compared to older (31–40 years) patients. *Hp* infection was more commonly seen in the Lauren's intestinal type and early pT stages (T1+T2). Independent prognostic factors for worse outcomes included higher serum CA 72–4, CA 125 levels, positive resection margin, and stage pIII–pIV tumors. The 5-year survival rate was significantly higher in patients with radical resection than those without.

GCs in young Chinese patients were prevalent in women with advanced stages but showed no significant differences in clinicopathology between FGC and SGC groups. High serum CA 72–4 and CA 125 levels may help identify patients with worse outcomes. Radical resection improved postresection survival.

## Abstract 2

Supplemental Digital Content is available in the text

## INTRODUCTION

At present, the incidence of gastric carcinoma (GC) has steadily decreased worldwide due to effective *Helicobacter pylori* (*Hp*) screening and treatment, as well as early detection by upper endoscopy.^1^ However, GC remains the third leading cause of cancer-related deaths in China.^2,3^ In general, GC occurs primarily in elderly patients aged ≥60 years; only ∼2.7% to 15% of patients are ≤40 years old,^4–6^ as the early-onset gastric cancer (EOGC).^7^ Previous studies show that EOGCs occur mainly in female patients with the histology diffuse type (Lauren's classification), advanced tumor stage, and high incurable rates.^4,8,9^

Although detailed pathogenesis mechanisms of GC remain elusive, environmental factors combined with specific genetic alterations in the vulnerable population play critical roles in GC tumorigenesis.^10–12^ Because EOGC patients expose to environmental toxins much lesser than older patients, hereditary factors may be of more importance in EOGC tumorigenesis.^13^ Indeed, ∼10% to 25% of EOGC patients have a positive family history,^6,9,14,15^ some of whom have hereditary GC syndromes, such as hereditary diffuse GC with 25% to 50% of cases harboring germline *CDH1* gene mutations. However, the *CDH1* gene mutation rate differs considerably between high- and low-incidence regions in the world. In a Japanese study on *CDH1* gene mutations in 13 familial gastric cancer (FGC) families,^16^ only 1 missense somatic mutation was identified. Most Chinese studies also revealed no truncating germline *CDH1* gene mutations in FGC families or EODGC patients,^17–19^ a feature different from that reported in Europeans.^20,21^

The reports on prognosis of EOGC patients after resection also show conflicting results.^5,8,9,22–25^ Some studies demonstrate an unfavorable prognosis in very young (<30 years) GC patients, which was interpreted as a result of delayed diagnosis and rapid disease progression,^5,25^ whereas others report no significant differences in survival between the very young and older GC patient groups.^9,22–24^ Some investigators have even described better survival rates in EOGC patients, compared to older GC patients.^8^ However, few studies have focused on clinicopathologic features of young Chinese GC patients and those with a family history of GC in the first- or second-degree relatives. The aims of this study were to characterize clinicopathology of EOGC, compared to old (>41 years) GCs, elucidate prognostic factors, especially in familial EOGC patients, and compare differences between very young (≤30 years) and older (31–40 years) EOGC patients groups in a homogeneous Chinese population.

## METHODS

### Patients

We searched GC resection cases in the electronic pathology databank stored in the Department of Pathology of the Nanjing Drum Tower Hospital over the period between January 2004 and December 2014. All pathology reports were retrieved and reviewed by 2 experienced pathologists. Inclusion criteria for the EOGC study were: (1) surgical or endoscopic GC resection, (2) patient age ≤40 years. Exclusion criteria consisted of: (1) GC diagnosed by endoscopic biopsy without resection, (2) no archival tissue blocks available for recuts, and (3) the patient lost to follow-up. The control group consisted of 250 older (>41 years) GC patients recruited from the same study period. Consent for GC resection and research was obtained from each patient before the resection procedure was taken place. The study protocol was approved by the Medical Ethics Committee of the Nanjing Drum Tower Hospital.

### Data

Clinical features of each patient, extracted from the medical record, included demographics, symptoms and durations, body mass index (BMI), endoscopic findings (tumor site, macroscopic pattern defined by the WHO classification),^26^ adjuvant therapy, tumor recurrence, and *Hp* infection status (determined by the rapid urease test). Grossly, early GC was categorized as protruded (type 0–I), superficially elevated (type 0–IIa), superficially flat (type 0–IIb), superficially depressed (type 0–IIc), and excavated (type 0–III) patterns, whereas advanced GC showed 4 patterns, including polypoid (type I), fungating (type II), ulcerated (type III), and flat infiltrative feature (type IV). Tabulated were laboratory test results on serum levels of albumin (normal range: 35–50 g/L), CA 72–4 (normal range: 0–6.9 U/mL), CA 125 (normal range: 0–30.2 U/mL), CA 19–9 (normal range: 0–39 U/mL), CA 242 (normal range: 0–15 U/mL), CEA (normal range: 0–10 ng/mL), and AFP (normal range: 0–10 ng/mL). Also analyzed were surgical resection methods, operative morbidity, and pathological details such as tumor size and 4 primary tumor locations: (1) proximal, including gastroesophageal junction and proximal third of the stomach, (2) middle (corpus), and (3) distal stomach, from the incidura, antrum to pylorus, and (4) whole stomach. Pathology features assessed were Lauren's classification, the WHO tumor histology type, WHO tumor differentiation, tumor stage (based on the 7th edition of the American Joint Committee on Cancer [AJCC7]),^27^ lymphovascular invasion (LVI), perineural invasion (PNI) (defined as the process of neoplastic invasion of nerves),^28^ and resection margin status. All selected patients were interviewed and followed-up through telephone or home visit by a trained gastroenterologist for detailed family history (living status of first- and second-degree relatives of the proband). According to the FGC diagnostic guideline of the Netherland Research Group,^29^ the criteria for FGC included GC in 2 or more first- or second-degree relatives, with at least 1 having GC diagnosed before the age of 50 years or GC in 3 or more first- or second-degree relatives, independent of age. GC patients without a positive family history were grouped as spontaneous GC (SGC).

### Statistical Analysis

Categorical variables were compared with Pearson's Chi-square (χ^2^) test or Fisher's exact test; continuous variables were evaluated by Student's *t* test or Mann–Whitney *U* test. Correlations between various factors and *Hp* infection status were assessed by univariate and multivariate logistic regression analyses. Patient survival was estimated with the Kaplan–Meier method and a log-rank test. Postresection overall survival was analyzed by univariate and multivariate Cox proportional hazards regression models. Variables found to be statistically significant by the univariate analysis were further scrutinized backward stepwise by the multivariate analysis, in which the least significant variable was excluded sequentially. Independent risk factors were presented as the hazard ratio (HR) with 95% confidence interval (CI). All 2-tailed *P* values of <0.05 were considered statistically significant. All statistical analyses were performed using SPSS Statistics Version 22 (SPSS Inc, Chicago, IL).

## RESULTS

### Demographics and Baseline Characteristics

**FIGURE 1:**
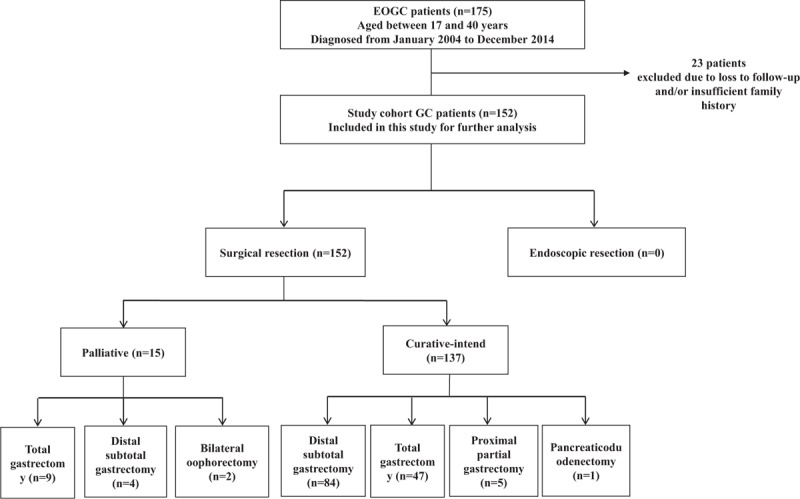
Flowchart showing early-onset gastric cancer study cohort and treatment modalities.

**FIGURE 2:**
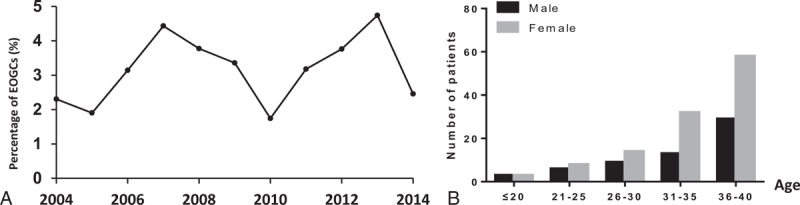
Percentage of EOGC patients treated annually and frequency of male vs female patients; (A) percentage of EOGC patients operated annually over the period from 2004 to 2014. (B) Number of male and female patients in different age groups. EOGC = early-onset gastric cancer.

Among 175 EOGC patients with the median age of 33 years (range: 17–40), 23 patients were lost to follow-up or did not have a detailed family history and thus excluded from the analysis (Figure 1). A total of 152 young patients and 250 old patients were eligible for the study (Table 1). The mean follow-up time for surviving patients was 38.1 months. There was no significant change in the trend in EOGC resections observed over the 11-year study period (Figure 2A). The overall male-to-female ratio was 0.53:1, which was significantly lower than that of old patients (*P* < 0.01, Table 1). The female-to-male patient ratio increased dramatically in the 31 to 40 years groups, compared to that of very young (≤ 30 years) patient group (Figure 2B).

**TABLE 1:**
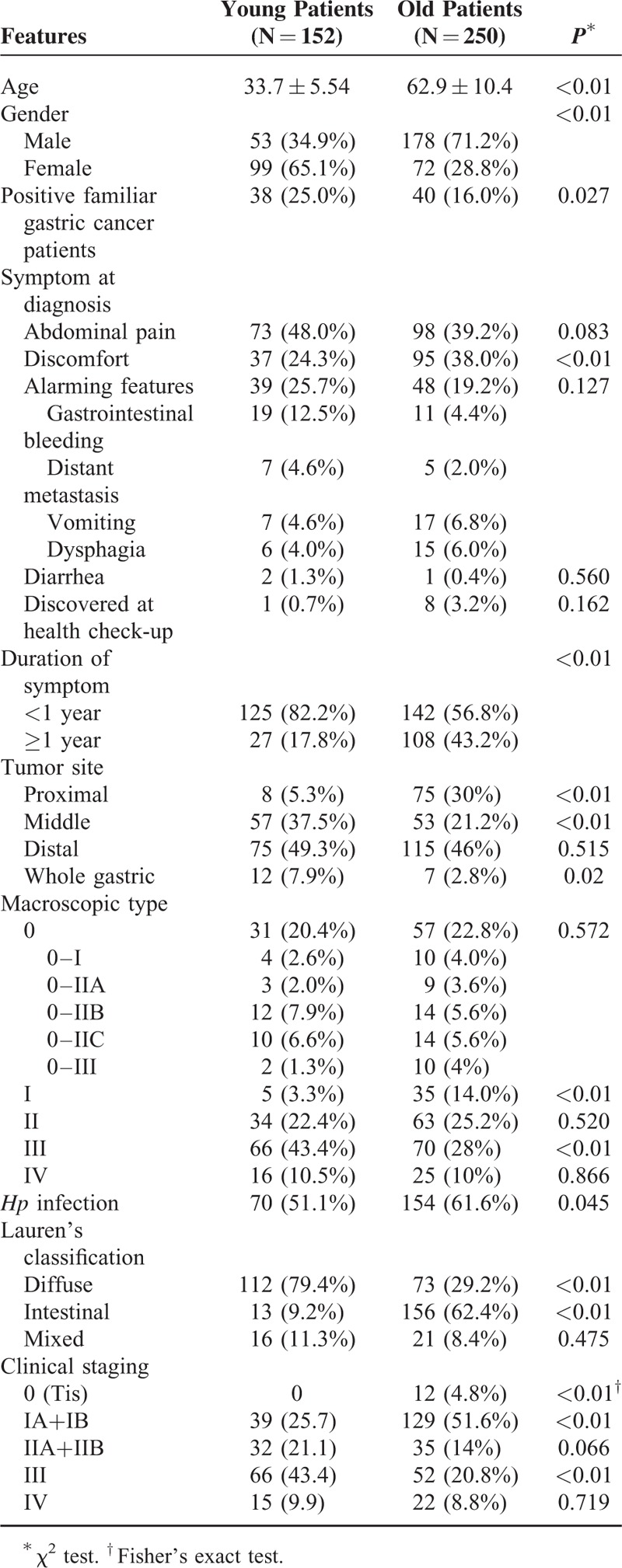
Clinicopathologic Features of Early-Onset Gastric Carcinoma in Chinese Patients (Younger than 40 Years of Age) Versus Those in Patients 41 Years or Older

### Clinical Findings

Overall, the symptom at diagnosis of all patients was nonspecific (Table 1). Only 25.7% EOGC and 19.2% old GC patients showed alarming clinical presentations, such as severe dysphagia, considerable gastrointestinal bleeding, and vomiting. Most young patients (82.2%) had a short duration of symptoms.

### Upper Endoscopy

The most common EOGC location was in the distal (49.3%) stomach, followed by the middle (37.5%). Only 5.3% of cases occurred in the proximal stomach (Table 1). In contrast in old patients, GC tumors were significantly more common in the proximal stomach (*P* < 0.01), but significantly less frequent in the middle (*P* < 0.01), and similarly common in the distal stomach (Table 1). Macroscopically in EOGCs, the most common pattern was type III (43.4%), followed by type II and type 0; the type I pattern was the least (3.3%) frequent, and whole stomach involvement was found in 16 patients (10.5%). In type 0 GCs, type IIB and IIC lesions were most common (7.9% and 6.6%). In comparison in old GCs, the type I pattern was significantly more common (*P* < 0.01) and the type III pattern was significantly less frequent (*P* < 0.05). Interestingly, the EOGC group had significantly lower frequent *Hp* infection rate than the old GC (*P* < 0.05, Table 1).

### Histopathology

According to Lauren's classification, the diffuse type (79.4%) was significantly more common in young GCs (*P* < 0.01), whereas the intestinal type was significantly more common in old GCs (62.4%) (*P* < 0.01). As shown in Table 1, 43.4% of young patients were significantly more frequently diagnosed at advanced stage (pIII, *P* < 0.01), compared with 20.8% in old patients. In EOGCs, tumor distant metastasis (n = 15) was limited to abdominal organs, mainly to the ovary (n = 6), peritoneum (n = 4), colon (n = 4), and pancreas (n = 1).

### Clinicopathological Characteristics of Familial EOGC

**FIGURE 3:**
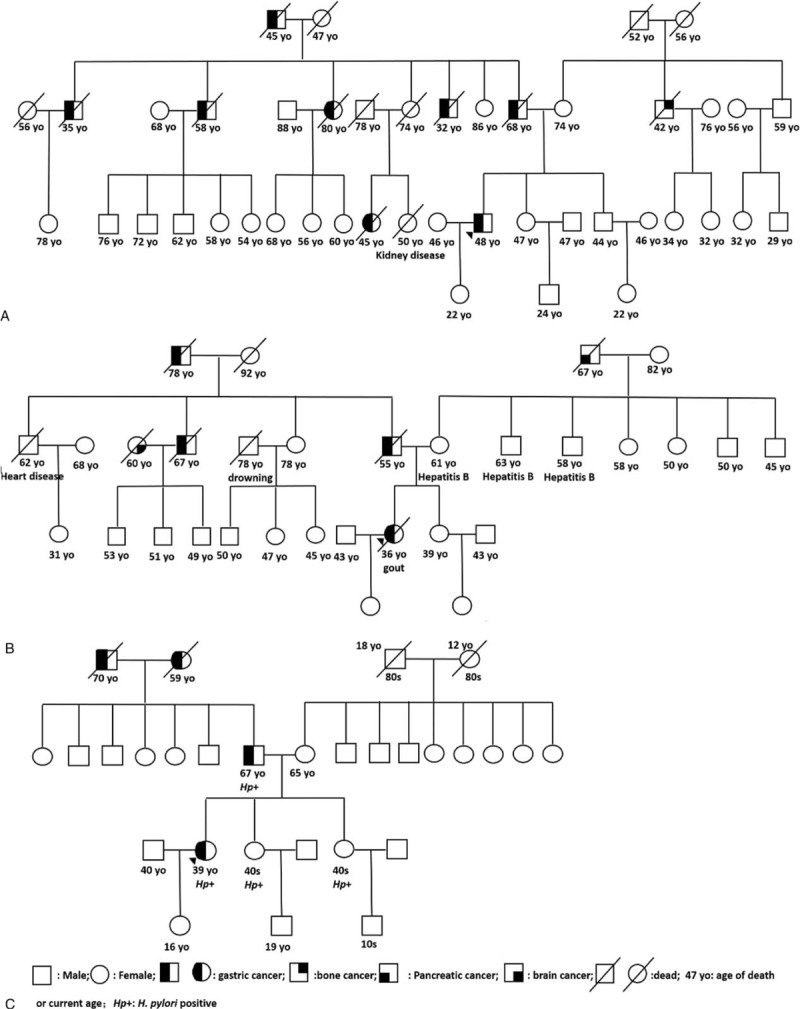
Typical pedigree of familiar gastric carcinoma patient.

Detailed family cancer history was available in all 152 patients (Table 2 and Figure 3). Thirty-eight (25%) GC cases met the FGC diagnostic criteria, whereas the majority (n = 114, 75%) were classified as SGC. Among typical pedigrees of FGC, the autosomal dominant hereditary pattern was most common. The most common organ with cancer in the relatives was, in the descending order, the stomach, esophagus, liver, lung, and colorectum (Table 3). In contrast, esophageal, hepatic, and pulmonary cancers were significantly more frequently found in the SGC group. However, the difference in overall clinicopathology between the 2 groups was not statistically significant.

**TABLE 2:**
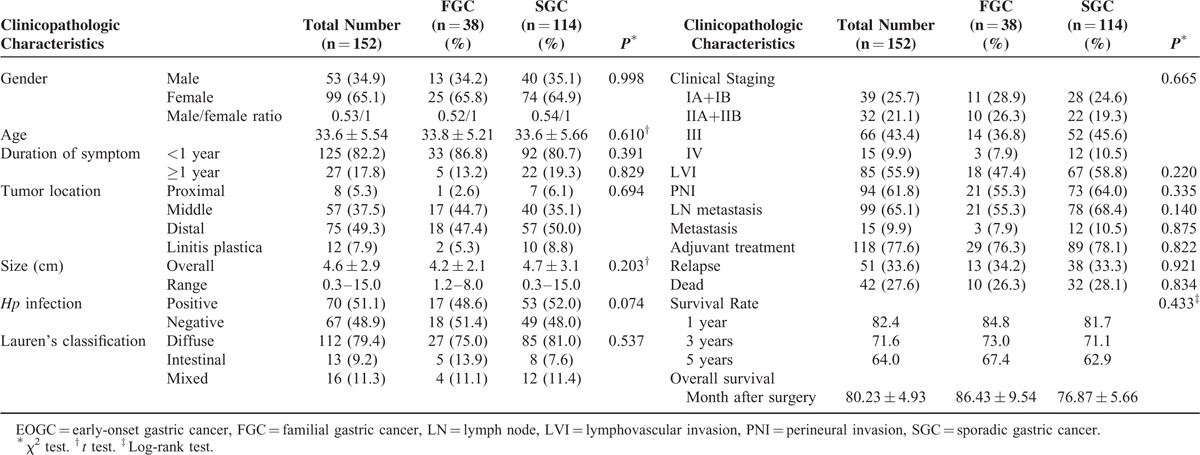
Comparison in Clinicopathology Features Between Familial and Sporadic EOGC Patients

**TABLE 3:**
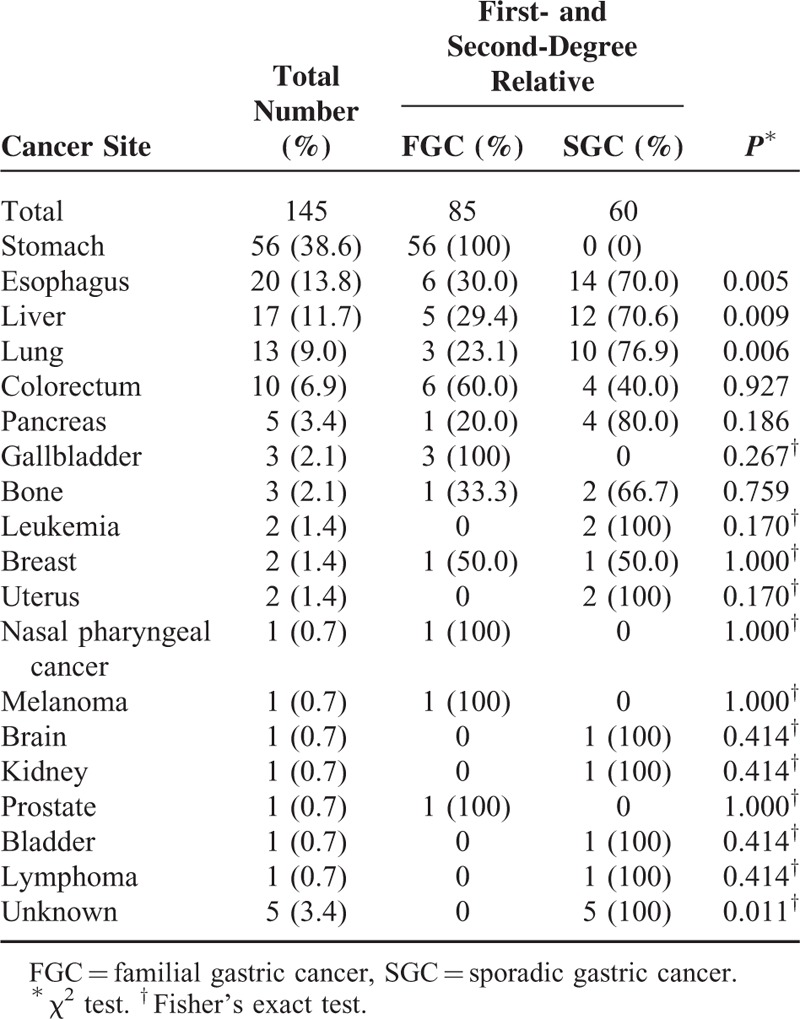
Cancers Among the First- and Second-Degree Relatives of Early-Onset Gastric Carcinoma Patients Between Familial and Sporadic Groups

### Differences in Clinicopathology between Very Young (≤30 years) and Older (31–40 years) EOGC Patient Groups

To investigate the biological behavior of EOGCs, we further divided the cohorts into 2 groups, according to age (Supplementary Table 1). The very young EOGC (≤30 years) group, compared to the older (31–40 years) group, was significantly more likely to have more frequent PNI (76.3% vs 57.0%, *P* = 0.034). No other significant differences in clinicopathology were found between the 2 groups.

### *Hp* Infection Status

*Hp* infection was detected in 51.1% of EOGC cases. The clinicopathological difference between *Hp*-positive and -negative patients was shown in Supplementary Table 2. Interestingly, the *Hp* infection rate was significantly higher in the older (31–40 years, 55.1%) GC group, Lauren's intestinal type, and longer symptom duration, but significantly lower in patients staged at pIII and pIV (*P* = 0.002) and PNI (*P* = 0.001). Consequently, the survival rate in patients with *Hp* infection was significantly higher than those without. Univariate logistic regression analysis showed that patient age, advanced macroscopic pattern (i.e. local or infiltrating, ulcerative, whole gastric involvement), Lauren's intestinal type, larger tumor size >4.0 cm, advanced tumor stage (cIII and cIV), pT stage, and PNI were significantly associated with *Hp* infection, whereas Lauren's intestinal type and early pT stage (pT1 and pT2) were the independent risk factors for *Hp* infection by multivariate analysis (Table 4).

**TABLE 4:**
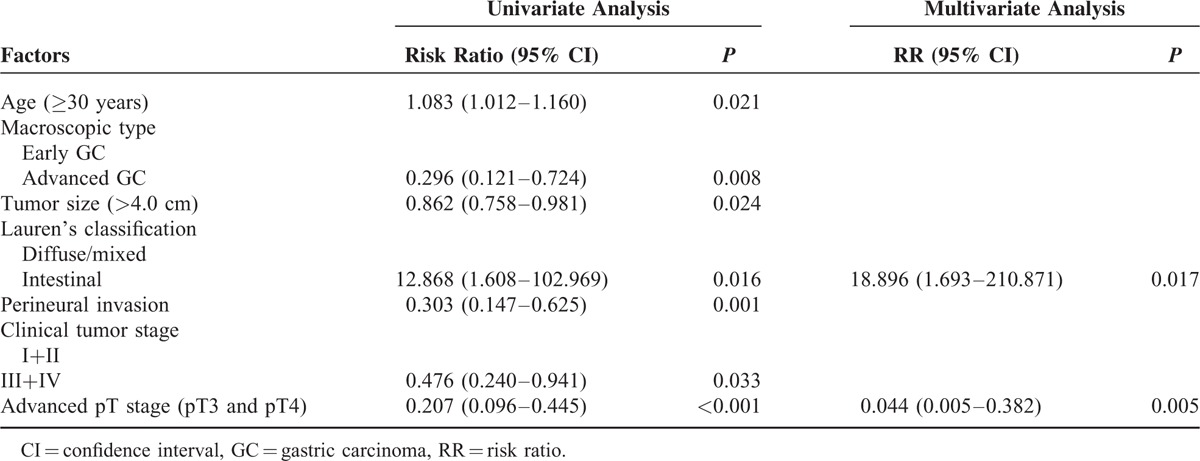
Univariate and Multivariate Analyses (Logistic Regression) of Clinicopathological Features in Relation to *Hp* Infection in Early-Onset Gastric Cancer Patients

### Prognostic Factors for Overall Survival

**FIGURE 4:**
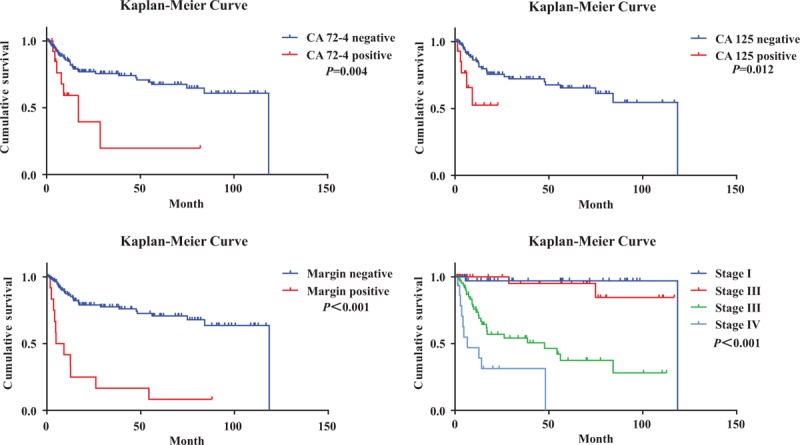
Kaplan–Meier curve showing the probability of overall survival for EOGC patients, according to CA 72–4 level, CA 125 level, resection margin, and pathology stage (log-rank test). EOGC = early-onset gastric cancer.

In the cohort, 137 EOGC patients (90.1%) underwent radical R0 surgical resection with curative intent. Tumor palliative resection was carried out in 15 (9.9%) (R1 resection) patients. Two EOGC patients underwent endoscopic submucosal dissection with subsequent additional open surgical resection with nodal dissection because of signet-ring cell carcinoma. One patient had severe postoperative complications with multiple organ failure and died of extensive abdominal metastasis. Of 152 EOGC patients, 42 (27.6%) died of cancer-specific causes. Univariate analysis (Table 5) identified poor prognostic factors including lower BMI (<18.5 kg/m^2^), the absence of *Hp* infection, lower serum levels of albumin, higher levels of CA 72–4, CA 125, and CA 19–9, larger tumor size of >4 cm, whole stomach involvement, advanced macroscopic patterns (type pIII–pIV),^30^ palliative R1 surgical resection, advanced pathologic stage (pIII and pIV), LVI, PNI, and resection margin involvement. By multivariate analysis (Table 5), independent worse prognostic factors included higher serum levels of CA 72–4 (HR: 7.673, 95% CI: 2.475–23.791, *P*<0.001), higher CA 125 (HR: 3.903, 95% CI: 1.121–13.590, *P* = 0.032), positive resection margin (HR: 11.081; 95% CI: 3.957–31.028, *P* = 0.017), and advanced tumor stage pIII (HR: 12.851), 95% CI: 1.601–103.122, *P* = 0.016) and pIV (HR: 72.516, 95% CI: 7.750–678.516, *P* <* *0.001). Kaplan–Meier survival curves for EOGC patients, according to independent prognostic factors, were exhibited in Figure 4.

**TABLE 5:**
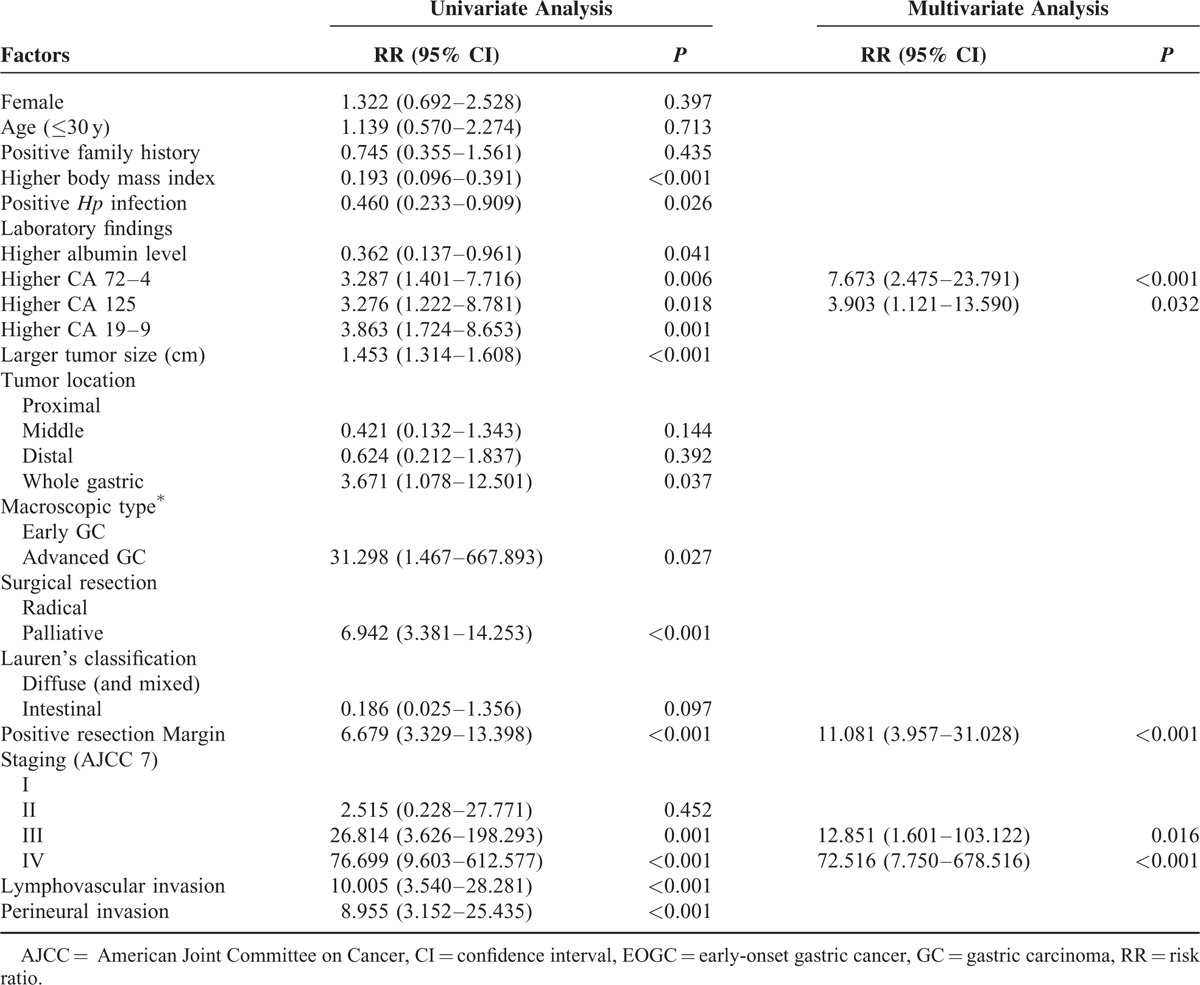
Uni- and Multivariate Analyses (Cox Regression) on Prognosis of EOGC Patients

## DISCUSSION

In line with previous similar investigations in other ethnic populations,^4,22,31,32^ our study reveals that EOGCs in Chinese patients treated at our hospital are also female predominant and show mainly the diffuse histology type. Although FGC accounts for only 25% of the EOGC cohort, there are no significant differences in clinicopathology between FGC and SGC groups. However, very young (≤ 30 years) patients have a significantly lower *Hp* infection rate, but a higher PNI rate than older patients, the findings that have not been described before.^33,34^ To our surprise, high serum levels of CA 72–4 and CA 125, along with palliative resection, are identified as independent risk factors for worse outcomes in EOGC patients, which may be clinically useful for patient management, if confirmed by additional clinical studies with larger samples from other centers.^35,36^

EOGC is uncommon and found in only 3.2% of all GC resections in our cohort, which is consistent with previous studies (2–8%).^15,37–39^ Although the true incidence of EOGC in China remains unknown, a recent American study, based on the data of the National Cancer Institute's Surveillance Epidemiology and End Results (SEER) program in the United States, shows a significantly increased incidence trend from 0.27 to 0.45 per 100,000 person-years for both female and male patients aged between 25 and 39 years.^40^ However, that study is limited to noncardiac GC only and carried out in a low GC risk population. In contrast, a significantly decreased EOGC incidence for male patients has been reported by Korean investigators under the Population-Based Regional Cancer Registry (PBCR) program in Korea,^41^ which is a high GC risk population, as the Chinese.

In general, family cancer aggregation is more common in EOGC patients than in older age GC groups, as also shown in our study. In our cohort, FGC accounted for 25% of EOGCs, a percentage similar to that reported by Umeyama et al in Japan,^6^ but slightly higher than that described by investigators from Shanghai in China^9^ and in Korea.^15,35^ Our studies show that the EOGC tumor location is more frequent in the middle stomach, which is consistent with that reported previously in Chinese^9^ and Japanese^42^ studies, suggesting underlying unique, probably hereditary, pathogenesis mechanisms that are different from those in the proximal or distal stomach. Nevertheless, the published data indicate an important role of hereditary factors in tumorigenesis of EOGC in both Western and Asian populations. Compared with SGC, the FGC group does show more frequent esophageal, liver and lung cancers in the proband first- and second-degree relatives. Interestingly, those cancers rank the highest in incidence among other organ types of cancer in the general population in China,^3^ suggesting a hereditary component(s) in those cancers. A recent genomic study^43^ points out that germline mutation of the *CDH1*, *CTNNA1,* or *MAP3K6* genes may be involved in the tumorigenesis of some FGCs. Further investigations are needed to reveal genomic mechanisms of hereditary GC in Chinese patients. In contrast in the SGC group, lung and liver, but not esophageal, cancers are predominant, a finding consistent with the data from the Chinese National Central Cancer Registry (NCCR) published in 2011.^3^ Apparently, EOGC in the SGC group may be part of common cancer syndromes but the hereditary relationship may exist between esophageal and gastric cancers in the Chinese population, as alluded by the most recent meta-analysis of GC genomics.^44^

GC is more common in men with a male/female ratio ranging from 1.62:1 to 2:1.^45,46^ However, most surveys on EOGC, including our own, have shown a female dominance with the male/female ratio ranging from 0.64:1 to 0.87:1.^15,35,47^ This observation suggests the potential role of estrogen in EOGC pathogenesis. According to a prospective study on Spanish women,^48^ the risk of GC increased in women who had oophorectomy, indicating the protective effect of estrogen against GC development, which has been confirmed by 2 large-scale studies in Japan^49^ and in China.^50^ Further investigation to illustrate molecular mechanisms by which estrogen plays in EOGC tumorigenesis is needed.

*Hp* infection has been proven to be carcinogenic in GC development but conveys a favorable survival outcome in GC patients with *Hp* infection, compared to those without *Hp* infection,^51^ which is also our experience in EOGC. Moreover, we show that very young (≤ 30 years) GC patients are less likely to be infected with *Hp* and less exposure to environmental toxins,^52^ suggesting that hereditary factors may be of more importance than *Hp* infection in tumorigenesis of EOGC. We showed that the absence of *Hp* infection was associated with a shorter symptom duration, more advanced tumor stage, and more frequent PNI, which are consistent with those reported in 1995 by a research group from Taiwan.^51^ Lee et al first reported that *Hp* seropositive GC patients with localized Borrmann types showed better survival than *Hp*-negative counterparts.^51^ Recently, *Hp* infection was found to be an independent prognostic factor for relapse-free survival and overall survival.^53,54^ The immune response activated by *Hp* infection could lead to genesis of gastric adenocarcinoma (activation of Th17 pathway),^55^ but meanwhile can also modulate antitumor immunity.^54^ The molecular interplay between *Hp* infection and host genetic vulnerability is essential for illustration of EOGC pathogenesis mechanism.

The postresection survival of EOGC patients remains elusive.^56^ In our study, the 5-year survival rate in EOGC patients was 64%, which is much higher than that reported by Korean and Japanese investigators.^9,35^ The discrepancy may be related to several factors. First, we included only the patients who underwent surgical or endoscopic resection with additional surgical nodal dissection. This indirectly suggests that if young GC patients diagnosed at a resectable stage, the prognosis would be favorable. Second, most patients in our cohort have undergone radical resection that demonstrates a significant survival advantage than those with palliative surgery only.

A major limitation of our study is the retrospective study design. As a result, not all cases have a complete dataset for analysis. In addition, we rely on patient self-reporting family history, which might have contributed to under-reporting of second-degree positive family history and under-diagnosed FGC. Although those variables are difficult to be controlled in the present study, we are currently conducting a robust prospective clinical investigation with a major focus on hereditary GC diseases at our center.

## CONCLUSION

We show that family aggregation in GC is more common in EOGC patients but FGC patients have clinicopathological features similar to SGC patients; early detection of high serum levels of CA 72–4 and CA 125 and subsequent radical, rather than palliative, resection could improve survival outcomes, especially for those with positive family history. Further genomic studies of EOGC especially FGC may help reveal molecular tumorigenesis mechanisms to provide EOGC patients with optimal individualized precision management strategy.

## Supplementary Material

Supplemental Digital Content

## Supplementary Material

Supplemental Digital Content
