# Supplementary material for: Gastric Carcinomas in Young (Younger than 40 Years) Chinese Patients: Clinicopathology, Family History, and Postresection Survival
Source: Medicine (Baltimore). 2016 Mar 7;95(9):e2873. doi: 10.1097/MD.0000000000002873 (PMC4782856; doi:10.1097/MD.0000000000002873)
Supplement: Supplemental Digital Content [file medi-95-e2873-s001.pptx]

## Slide 1
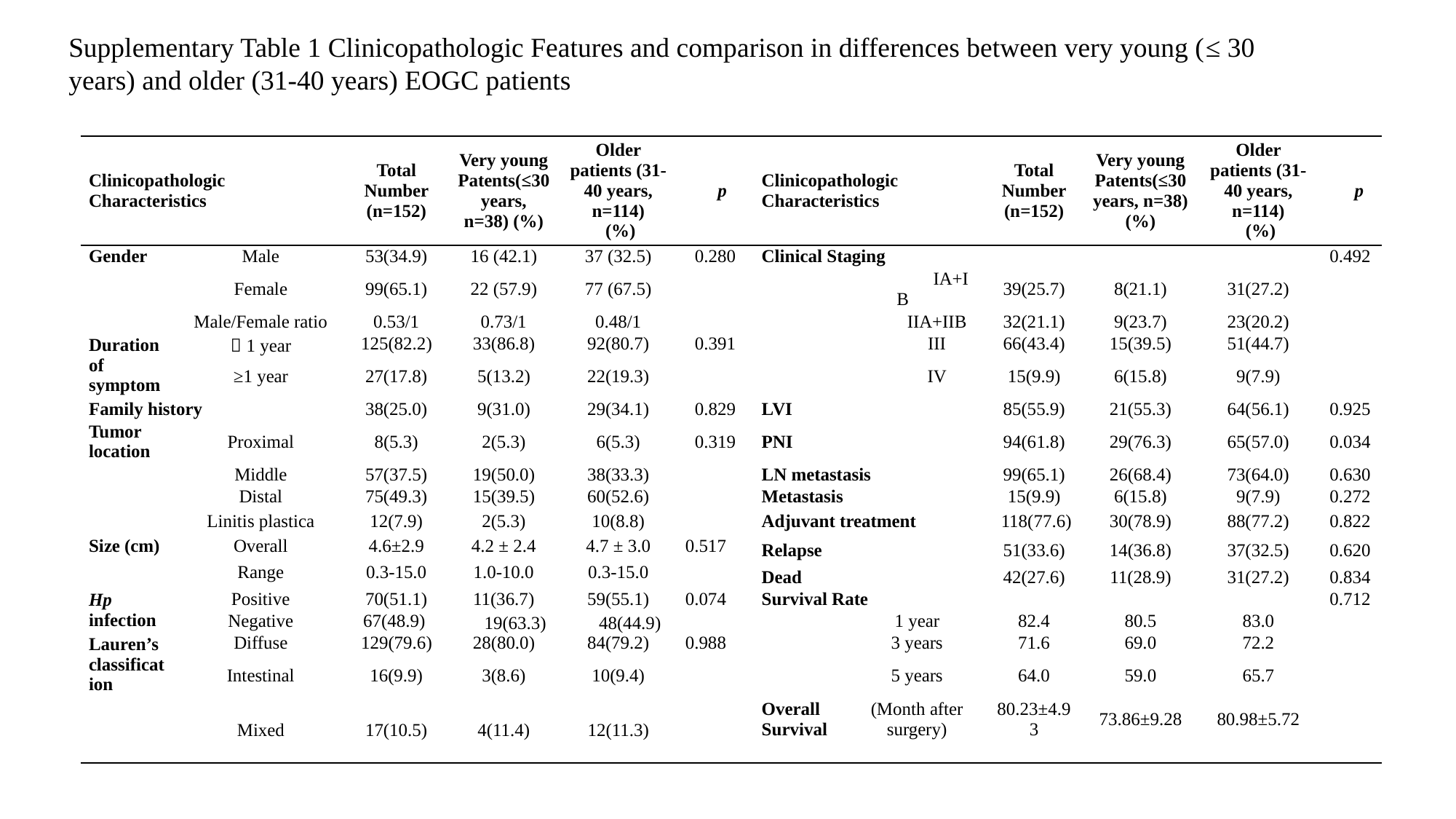

Supplementary Table 1 Clinicopathologic Features and comparison in differences between very young (≤ 30 years) and older (31-40 years) EOGC patients
| Clinicopathologic Characteristics | | Total Number (n=152) | Very young Patents(≤30 years, n=38) (%) | Older patients (31-40 years, n=114) (%) | p | Clinicopathologic Characteristics | | | Total Number (n=152) | Very young Patents(≤30 years, n=38) (%) | Older patients (31-40 years, n=114) (%) | p |
| --- | --- | --- | --- | --- | --- | --- | --- | --- | --- | --- | --- | --- |
| Gender | Male | 53(34.9) | 16 (42.1) | 37 (32.5) | 0.280 | Clinical Staging | | | | | | 0.492 |
| | Female | 99(65.1) | 22 (57.9) | 77 (67.5) | | | | IA+IB | 39(25.7) | 8(21.1) | 31(27.2) | |
| | Male/Female ratio | 0.53/1 | 0.73/1 | 0.48/1 | | | | IIA+IIB | 32(21.1) | 9(23.7) | 23(20.2) | |
| Duration of symptom | ＜1 year | 125(82.2) | 33(86.8) | 92(80.7) | 0.391 | | | III | 66(43.4) | 15(39.5) | 51(44.7) | |
| | ≥1 year | 27(17.8) | 5(13.2) | 22(19.3) | | | | IV | 15(9.9) | 6(15.8) | 9(7.9) | |
| Family history | | 38(25.0) | 9(31.0) | 29(34.1) | 0.829 | LVI | | | 85(55.9) | 21(55.3) | 64(56.1) | 0.925 |
| Tumor location | Proximal | 8(5.3) | 2(5.3) | 6(5.3) | 0.319 | PNI | | | 94(61.8) | 29(76.3) | 65(57.0) | 0.034 |
| | Middle | 57(37.5) | 19(50.0) | 38(33.3) | | LN metastasis | | | 99(65.1) | 26(68.4) | 73(64.0) | 0.630 |
| | Distal | 75(49.3) | 15(39.5) | 60(52.6) | | Metastasis | | | 15(9.9) | 6(15.8) | 9(7.9) | 0.272 |
| | Linitis plastica | 12(7.9) | 2(5.3) | 10(8.8) | | Adjuvant treatment | | | 118(77.6) | 30(78.9) | 88(77.2) | 0.822 |
| Size (cm) | Overall | 4.6±2.9 | 4.2 ± 2.4 | 4.7 ± 3.0 | 0.517 | Relapse | | | 51(33.6) | 14(36.8) | 37(32.5) | 0.620 |
| | Range | 0.3-15.0 | 1.0-10.0 | 0.3-15.0 | | | | | | | | |
| | | | | | | Dead | | | 42(27.6) | 11(28.9) | 31(27.2) | 0.834 |
| Hp infection | Positive | 70(51.1) | 11(36.7) | 59(55.1) | 0.074 | Survival Rate | | | | | | 0.712 |
| | Negative | 67(48.9) | 19(63.3) | 48(44.9) | | | 1 year | | 82.4 | 80.5 | 83.0 | |
| Lauren’s classification | Diffuse | 129(79.6) | 28(80.0) | 84(79.2) | 0.988 | | 3 years | | 71.6 | 69.0 | 72.2 | |
| | Intestinal | 16(9.9) | 3(8.6) | 10(9.4) | | | 5 years | | 64.0 | 59.0 | 65.7 | |
| | Mixed | 17(10.5) | 4(11.4) | 12(11.3) | | Overall Survival | (Month after surgery) | | 80.23±4.93 | 73.86±9.28 | 80.98±5.72 | |
| | | | | | | | | | | | | |
