# Supplementary material for: Gastric Carcinomas in Young (Younger than 40 Years) Chinese Patients: Clinicopathology, Family History, and Postresection Survival
Source: Medicine (Baltimore). 2016 Mar 7;95(9):e2873. doi: 10.1097/MD.0000000000002873 (PMC4782856; doi:10.1097/MD.0000000000002873)
Supplement: Supplemental Digital Content [file medi-95-e2873-s002.pptx]

## Slide 1
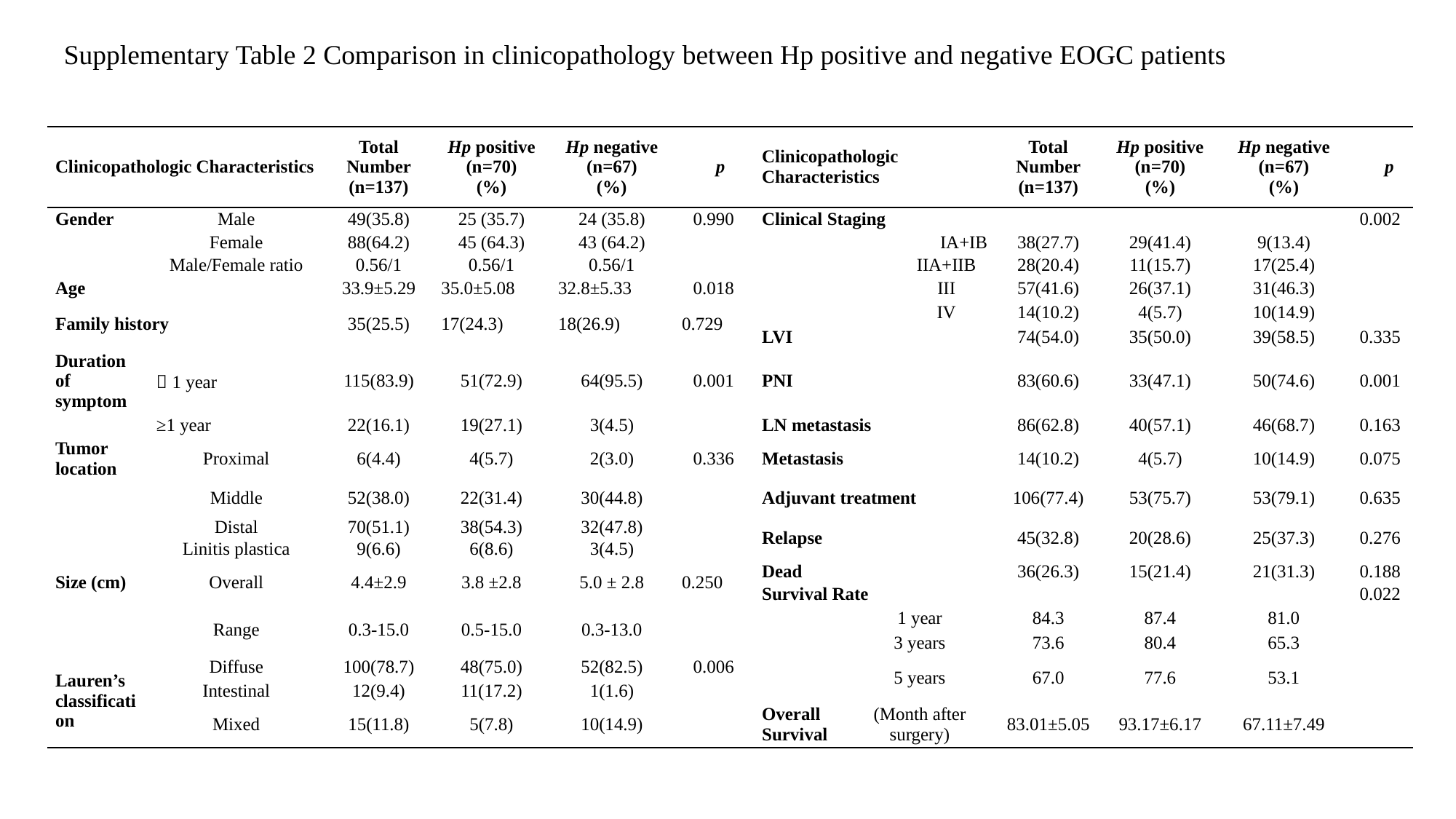

Supplementary Table 2 Comparison in clinicopathology between Hp positive and negative EOGC patients
| Clinicopathologic Characteristics | | Total Number (n=137) | Hp positive (n=70) (%) | Hp negative (n=67) (%) | p | Clinicopathologic Characteristics | | | Total Number (n=137) | Hp positive (n=70) (%) | Hp negative (n=67) (%) | p |
| --- | --- | --- | --- | --- | --- | --- | --- | --- | --- | --- | --- | --- |
| Gender | Male | 49(35.8) | 25 (35.7) | 24 (35.8) | 0.990 | Clinical Staging | | | | | | 0.002 |
| | Female | 88(64.2) | 45 (64.3) | 43 (64.2) | | | | IA+IB | 38(27.7) | 29(41.4) | 9(13.4) | |
| | Male/Female ratio | 0.56/1 | 0.56/1 | 0.56/1 | | | | IIA+IIB | 28(20.4) | 11(15.7) | 17(25.4) | |
| Age | | 33.9±5.29 | 35.0±5.08 | 32.8±5.33 | 0.018 | | | III | 57(41.6) | 26(37.1) | 31(46.3) | |
| Family history | | 35(25.5) | 17(24.3) | 18(26.9) | 0.729 | | | IV | 14(10.2) | 4(5.7) | 10(14.9) | |
| | | | | | | LVI | | | 74(54.0) | 35(50.0) | 39(58.5) | 0.335 |
| Duration of symptom | ＜1 year | 115(83.9) | 51(72.9) | 64(95.5) | 0.001 | PNI | | | 83(60.6) | 33(47.1) | 50(74.6) | 0.001 |
| | ≥1 year | 22(16.1) | 19(27.1) | 3(4.5) | | LN metastasis | | | 86(62.8) | 40(57.1) | 46(68.7) | 0.163 |
| Tumor location | Proximal | 6(4.4) | 4(5.7) | 2(3.0) | 0.336 | Metastasis | | | 14(10.2) | 4(5.7) | 10(14.9) | 0.075 |
| | Middle | 52(38.0) | 22(31.4) | 30(44.8) | | Adjuvant treatment | | | 106(77.4) | 53(75.7) | 53(79.1) | 0.635 |
| | Distal | 70(51.1) | 38(54.3) | 32(47.8) | | Relapse | | | 45(32.8) | 20(28.6) | 25(37.3) | 0.276 |
| | Linitis plastica | 9(6.6) | 6(8.6) | 3(4.5) | | | | | | | | |
| Size (cm) | Overall | 4.4±2.9 | 3.8 ±2.8 | 5.0 ± 2.8 | 0.250 | Dead | | | 36(26.3) | 15(21.4) | 21(31.3) | 0.188 |
| | | | | | | Survival Rate | | | | | | 0.022 |
| | Range | 0.3-15.0 | 0.5-15.0 | 0.3-13.0 | | | 1 year | | 84.3 | 87.4 | 81.0 | |
| | | | | | | | 3 years | | 73.6 | 80.4 | 65.3 | |
| Lauren’s classification | Diffuse | 100(78.7) | 48(75.0) | 52(82.5) | 0.006 | | 5 years | | 67.0 | 77.6 | 53.1 | |
| | Intestinal | 12(9.4) | 11(17.2) | 1(1.6) | | | | | | | | |
| | Mixed | 15(11.8) | 5(7.8) | 10(14.9) | | Overall Survival | (Month after surgery) | | 83.01±5.05 | 93.17±6.17 | 67.11±7.49 | |
